# Bisphenol A Analogues Inhibit Human and Rat 11β-Hydroxysteroid Dehydrogenase 1 Depending on Its Lipophilicity

**DOI:** 10.3390/molecules28134894

**Published:** 2023-06-21

**Authors:** Hong Wang, Jianmin Sang, Zhongyao Ji, Yang Yu, Shaowei Wang, Yang Zhu, Huitao Li, Yiyan Wang, Qiqi Zhu, Renshan Ge

**Affiliations:** 1 Department of Anesthesiology and Perioperative Medicine, The Second Affiliated Hospital and Yuying Children’s Hospital of Wenzhou Medical University, Wenzhou 325027, Chinayywang0119@wmu.edu.cn (Y.W.); 2 Key Laboratory of Pediatric Anesthesiology, Ministry of Education, Wenzhou 325027, China; 3 Key Laboratory of Anesthesiology of Zhejiang Province, Wenzhou Medical University, Wenzhou 325027, China; 4 Key Laboratory of Structural Malformations in Children of Zhejiang Province and Key Laboratory of Male Health and Environment of Wenzhou, Wenzhou 325000, China; 5 Department of Obstetrics and Gynecology, The Second Affiliated Hospital and Yuying Children’s Hospital of Wenzhou Medical University, Wenzhou 325027, China

**Keywords:** bisphenol analog, glucocorticoid, 11β-hydroxysteroid dehydrogenase 1, cortisol, docking analysis, species difference

## Abstract

Bisphenol A (BPA) analogues substituted on the benzene ring are widely used in a variety of industrial and consumer materials. However, their effects on the glucocorticoid-metabolizing enzyme 11β-hydroxysteroid dehydrogenase 1 (11β-HSD1) remain unclear. The inhibitory effects of 6 BPA analogues on the inhibition of human and rat 11β-HSD1 were investigated. The potencies of inhibition on human 11β-HSD1 were bisphenol H (IC_50_, 0.75 µM) > bisphenol G (IC_50_, 5.06 µM) > diallyl bisphenol A (IC_50_, 13.36 µM) > dimethyl bisphenol A (IC_50_, 30.18 µM) > bisphenol A dimethyl ether (IC_50_, 33.08 µM) > tetramethyl bisphenol A (>100 µM). The inhibitory strength of these chemicals on rat 11β-HSD1 was much weaker than that on the human enzyme, ranging from 74.22 to 205.7 µM. All BPA analogues are mixed/competitive inhibitors of both human and rat enzymes. Molecular docking studies predict that bisphenol H and bisphenol G both bind to the active site of human 11β-HSD1, forming a hydrogen bond with catalytic residue Ser170. The bivariate correlation of IC_50_ values with LogP (lipophilicity), molecular weight, heavy atoms, and molecular volume revealed a significant inverse regression and the correlation of IC_50_ values with ΔG (low binding energy) revealed a positive regression. In conclusion, the lipophilicity, molecular weight, heavy atoms, molecular volume, and binding affinity of a BPA analogue determine the inhibitory strength of human and rat 11β-HSD isoforms.

## 1. Introduction

Bisphenols are a group of chemical compounds that are widely used in the production of plastics, resins, and other materials. Among the most commonly used bisphenols is bisphenol A (BPA). Bisphenols have been in use for decades and can be found in a wide range of products, including food packaging, water bottles, medical devices, and electronics [1].

However, concerns about the potential health effects of bisphenols have led to increased scrutiny of their use. Bisphenols have been identified as endocrine-disrupting chemicals (EDCs), meaning that they have the ability to interfere with the body’s hormonal systems [1,2,3,4]. This interference can lead to a variety of health problems, including reproductive disorders, developmental issues, and cancer [1,2,3,4].

One of the most well-known bisphenols is BPA, which has been extensively studied for its potential health effects. BPA has been linked to a variety of health problems, including breast and prostate cancer, obesity, and fertility issues [1,2,3,4].

However, due to its potential harmful effects on human health, regulators have been increasingly concerned about the use and exposure of BPA [1,5,6]. Studies have shown that BPA exposure can lead to various health problems, including reproductive disorders, obesity, type 2 diabetes, cardiovascular disease, and cancer [1,3,7]. Therefore, some countries have banned the use of BPA in certain consumer products.

Despite these bans and restrictions, BPA continues to be used in many consumer products. In addition, BPA exposure can occur not only via ingestion from food and beverage containers but also via dermal contact and inhalation [1,3,7].

However, as awareness of the potential health risks associated with BPA has grown, manufacturers have begun to replace it with other BPA analogs, such as 4,4′-(propane-2,2-diyl) bis (methoxy benzene) (BPAME) and 2,2-bis(2-hydroxy-5-biphenylyl) propane (also called bisphenol PH, BPH). While these analogs were initially thought to be safer alternatives to BPA, recent research has raised concerns about their potential health effects as well.

According to ECHA, there are over 60 BPA analogues, including one group of potential alternatives to BPA with substitutions on the benzene ring. These compounds contain either alkyl groups, such as methyl (dimethyl bisphenol A, DMBPA; tetramethyl bisphenol A, TMBPA), propyl bisphenol A (also called bisphenol G, BPG), or allyl (diallyl bisphenol A, DABPA), or aryl groups, such as aryl bisphenol A (BPH) [8,9,10]. BPA analogues are also formed by the ether, such as BPA dimethyl ether (BPAME) on the 4-hydroxyl groups of BPA. The detailed information on BPA analogues is provided in Supplementary Table S1, and their structures are presented in Table 1. Studies have shown that some BPA analogues have higher estrogenic activity compared to BPA [11,12]. However, it is important to understand the potential risks of these analogues before their widespread use.

**Figure 1:**
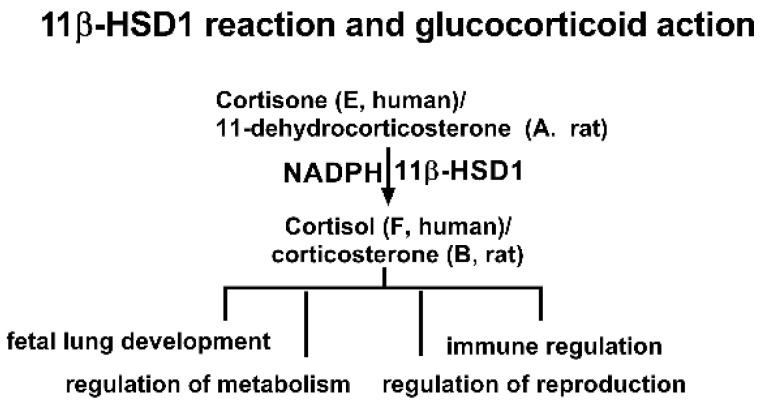
Scheme of 11β-hydroxysteroid dehydrogenase 1 (11β-HSD1) reaction and glucocorticoid action.

One potential target of BPA is its effects on glucocorticoid metabolism. Glucocorticoids, such as cortisol (F) in humans and corticosterone (CORT) in rats, are corticosteroid hormones that bind to the glucocorticoid receptor, a nuclear receptor that regulates gene expression [13,14,15]. Glucocorticoids are crucial for regulating stress responses, immune function, and metabolism [13]. The activity of glucocorticoids is tightly controlled by the enzyme 11β-hydroxysteroid dehydrogenase 1 (11β-HSD1), which converts inactive cortisone (E) to active F in humans or 11-dehydrocorticosterone (DHC) to CORT in rats (Figure 1) [16]. 11β-HSD1 is primarily present in glucocorticoid-target tissues such as the liver, gonad, lung, and fat [16]. Dysregulation of the glucocorticoid system by inhibiting 11β-HSD1 can lead to a range of diseases, including glucocorticoid deficiency, delayed fetal lung development, and immune system disorders [16]. Our previous study indicates that BPA is a weak inhibitor of 11β-HSD1 [17]. However, the toxicities of these new BPA analogues on 11β-HSD1 and the structure-activity relationship (SAR) remain unclear.

The 11β-HSD1 enzyme belongs to the short-chain dehydrogenase/reductase (SDR) family, with the catalytic motif Ser/Thr…Tyr-X-X-X-Lys residues [16]. For human 11β-HSD1, this catalytic domain is Ser170…Tyr179-X-X-X-Lys183, while for the rat 11β-HSD1, this domain is S166…Tyr173-X-X-X-Lys179 [16]. The catalytic reduction in 11β-HSD1 requires cofactor NADPH for assistance (Figure 1). The reductive direction of 11β-HSD1 is determined by the intraluminal microsomal hexose-6-phosphate dehydrogenase, which uses glucose-6-phosphate (G6P) to generate NADPH, deriving 11β-HSD1 toward reductive catalysis [18]. In this study, the inhibitory potency and mode of action of six BPA analogues on human and rat 11β-HSD1 were examined, and in silico molecular docking analyses were selected for understanding their underlying mechanisms.

## 2. Results

### 2.1. BPA Analogues Inhibit Human 11β-HSD1A Activity

**Figure 2:**
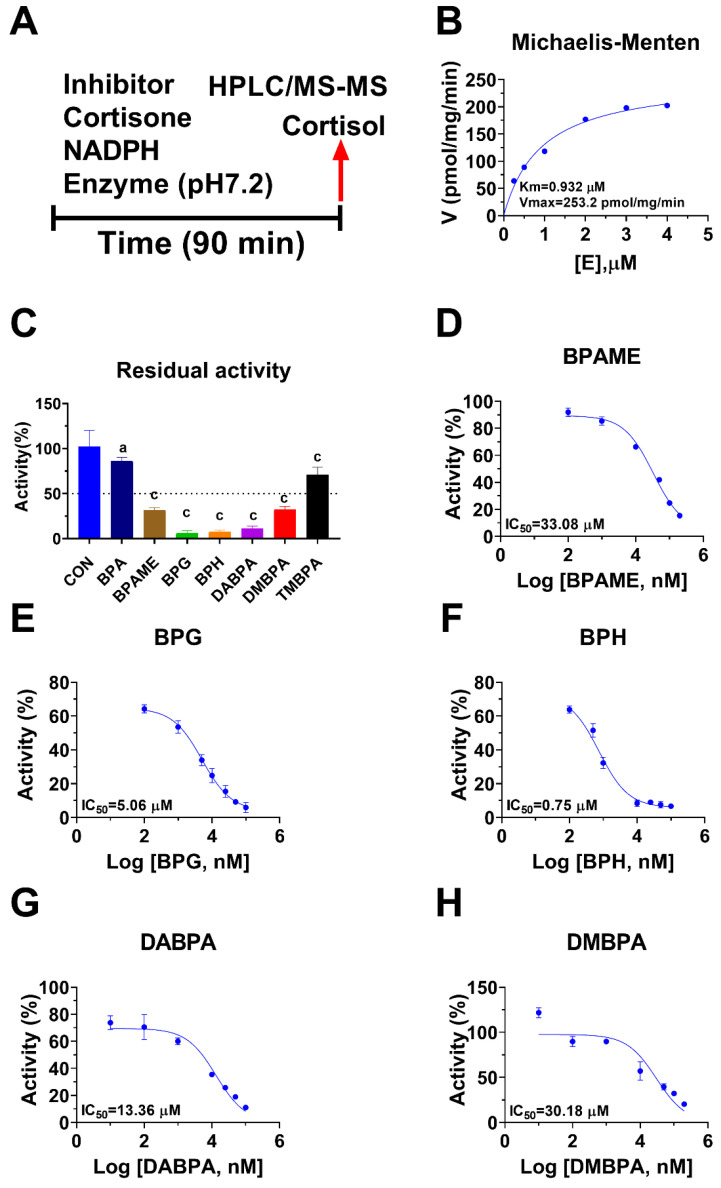
Regimen of assay, Michaelis-Menten kinetics and the effects of bisphenol A (BPA) analogues on enzymatic activity at 100 µM, and dose response in human liver 11β-hydroxysteroid dehydrogenase 1 (11β-HSD1). Regimen of assay (**A**); Michaelis-Menten kinetics (**B**), showing Km and Vmax; 11β-HSD1 residual activity relative to the control (DMSO) after BPA analogues at 100 µM compared with the control (**C**), ^a^ *p* < 0.05, ^c^ *p* < 0.001 (*n* = 4–6, mean ± SEM); IC_50_ values of (BPAME, BPG, BPH, DABPA, DMBPA) (Mean ± SEM, *n* =4), respectively (**D**–**H**).

**Figure 3:**
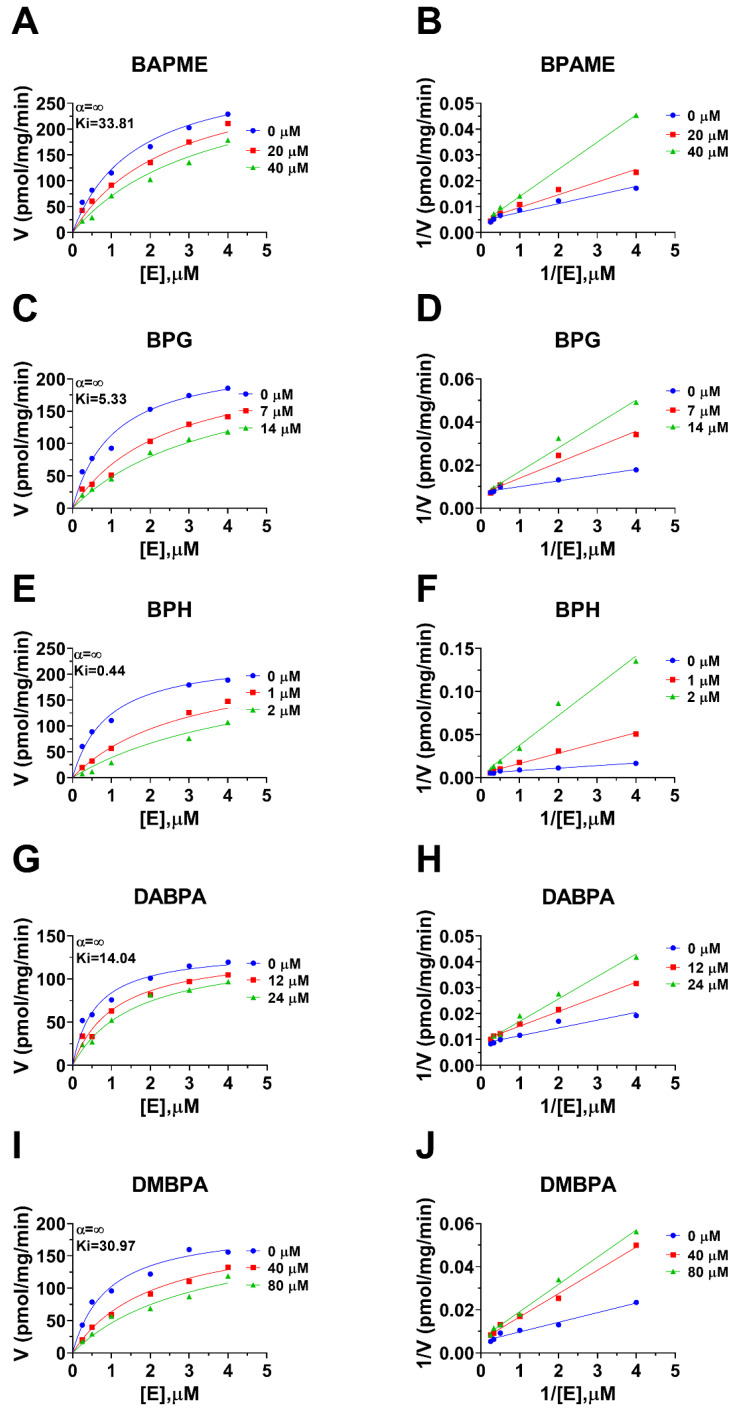
Enzyme kinetics–inhibition (mixed model) and Lineweaver-Burk plot of bisphenol A (BPA) analogues on human liver 11β-HSD1. Enzyme kinetics–inhibition analysis (**A**,**C**,**E**,**G**,**I**), Lineweaver-Burk plot (**B**,**D**,**F**,**H**,**J**): BPAME, BPG, BPH, DABPA, and DMBPA, respectively; Mean ± SEM, *n* = 4.

Human 11β-HSD1 in liver microsomes catalyzes the conversion of cortisone to cortisol (Figure 2A); the Km was 0.93 ± 0.22 µM (Figure 2B), which agreed with a previous report [19]. The Vmax of human 11β-HSD1 was 253.2 ± 20.5 pmol/mg/min (Figure 2B). BPA and six BPA analogues were examined to inhibit human 11β-HSD1 at 100 µM. BPA and TMBPA significantly inhibited the enzyme activity, but they led to residual activity greater than 50% (Figure 2C). However, BPAME, BPG, BPH, DABPA, and DMBPA at 100 µM significantly inhibited the enzyme activity, leading to residual activity of ≤50% (Figure 2C). Dose response analysis showed that IC_50_ values of BPA and BPA analogues ranged from 0.78 to 319.16 µM (Figure 2D–H and Table 2). The Ki values of BPA analogues ranged from 0.44 to 33.81 µM for BPAME, BPG, BPH, DABPA, and DMBPA, respectively (Figure 3 and Table 2). These results indicate that the potency of BPA and BPA analogues to inhibit human 11β-HSD1 shows structure-dependent differences. The α values indicated that BPAME, BPG, BPH, DABPA, and DMBPA are competitive inhibitors, suggesting that they bind to the free enzyme. The Lineweaver-Bruk plots confirmed their mode of action (Figure 3).

### 2.2. BPA Analogues Inhibit Rat 11β-HSD1 Activity

**Figure 4:**
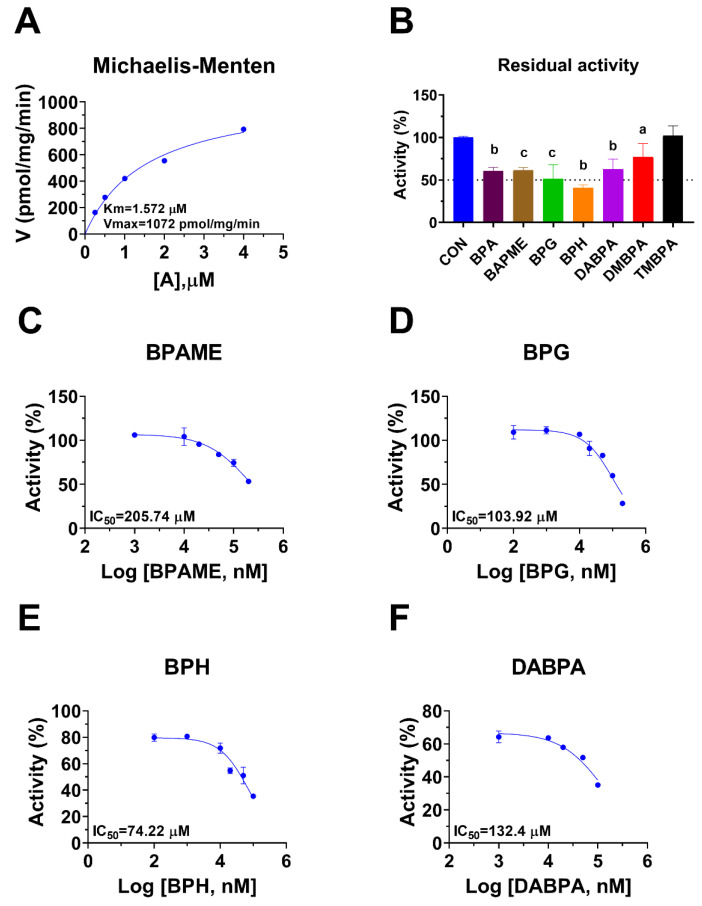
Michaelis-Menten kinetics, the effects of bisphenol A (BPA) analogues on enzymatic activity at 100 µM, and dose response in rat liver 11β-hydroxysteroid dehydrogenase 1 (11β-HSD1). Michaelis-Menten kinetics (**A**), showing Km and Vmax; 11β-HSD1 residual activity relative to the control (DMSO) after BPA analogues at 100 µM (**B**): compared with the control, ^a^ *p* < 0.05, ^b^ *p* < 0.01, ^c^ *p* < 0.001 (*n* = 4–6, mean ± SEM); IC_50_ values of BPAME, BPG, BPH, and DABPA (*n* = 4), respectively (**C**–**F**).

**Figure 5:**
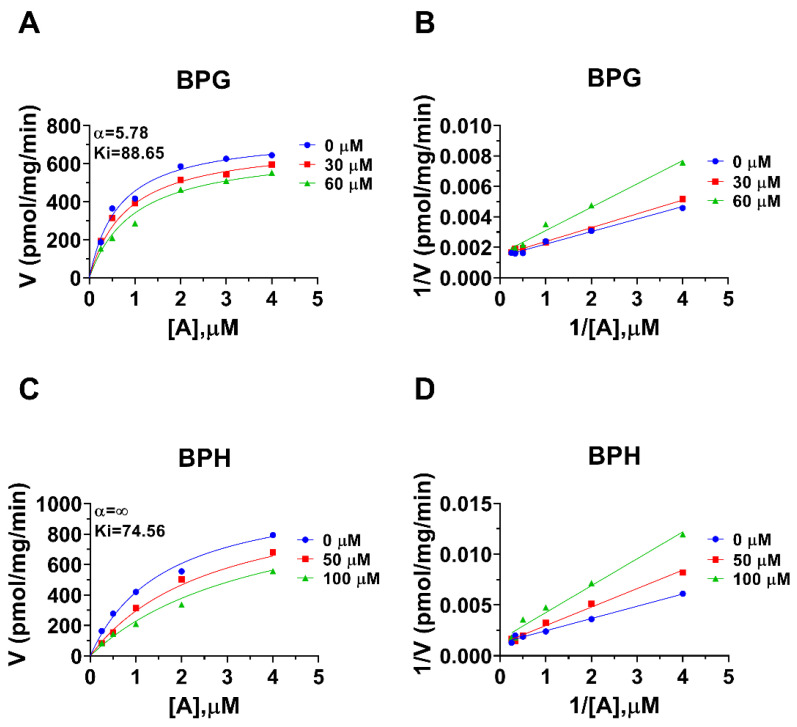
Enzyme kinetics–inhibition (mixed model) and Lineweaver-Burk plot of bisphenol A (BPA) analogues on rat liver 11β-HSD1. Enzyme kinetics–inhibition analysis (**A**,**C**), Lineweaver-Burk plot (**B**,**D**): BPG, BPH, respectively; Mean ± SEM, *n* = 4.

Rat 11β-HSD1 in liver microsomes catalyzes the conversion of DHC to CORT, and Km was 0.94 ± 0.09 µM (Figure 4A), which was in line with the reported value [20]. The Vmax of rat microsomal 11β-HSD1 was 1072 ± 115.5 pmol/mg/min (Figure 4A). BPA and six BPA analogues were examined to inhibit rat 11β-HSD1 at 100 µM. BPAME, DABPA, and DMBPA significantly inhibited the enzyme activity, but it led to residual activity greater than 50% (Figure 4B). BPA, BPG, and BPH at 100 µM significantly inhibited the enzyme activity, leading to a residual activity of ≤50% (Figure 4B). However, TMBPA at 100 µM was ineffective (Figure 4B). Dose response analysis showed that the IC_50_ values of BPA and BPA analogues ranged from 36.76 to 205.74 µM (Figure 4D–F and Table 2). The Ki values of BPG and BPH were 106.9 and 74.56 µM, respectively (Figure 5 and Table 2). These results indicate that the potency of BPA and BPA analogues to inhibit rat 11β-HSD1 shows structure-dependent differences. The α values indicated that BPG and BPH are mixed/competitive inhibitors, suggesting that they either bind to the free enzyme or the enzyme complex. The Lineweaver-Bruk plots confirmed their mode of action (Figure 5).

### 2.3. Molecular Docking Analysis of BPA Analogues with Human 11β-HSD1

**Figure 6:**
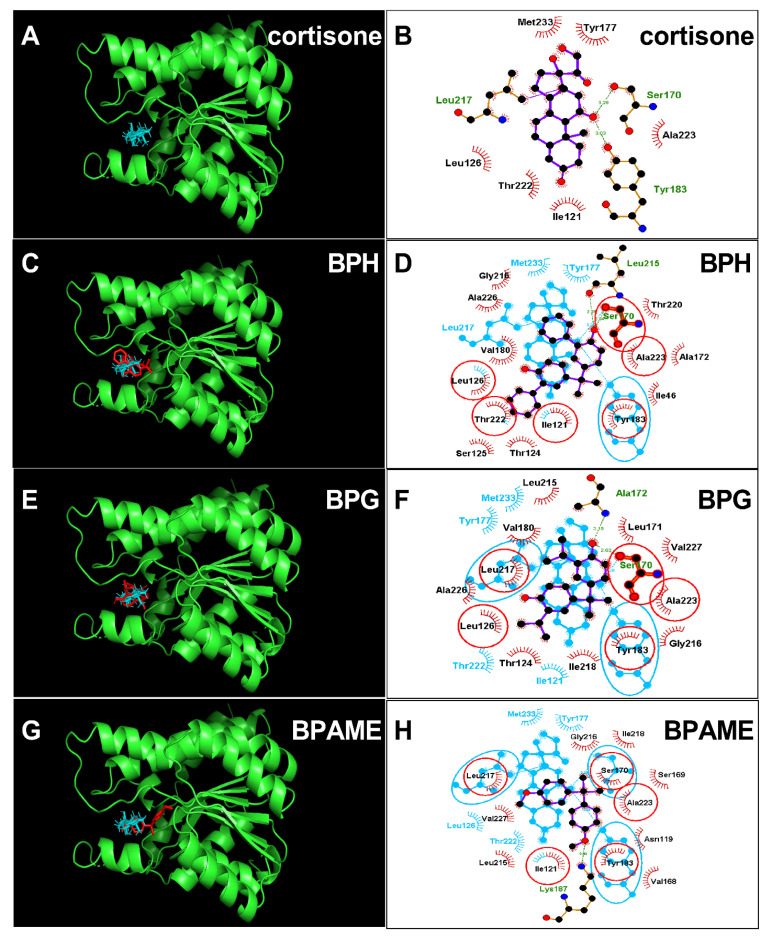
Molecular docking of cortisone, bisphenol H (BPH), bisphenol G (BPG) with human 11β-HSD1. Superimposed 3D structure models (BPA analogues = red; cortisone = cyan): Cortisone (**A**), BPH (**C**), BPG (**E**), BPAME (**G**); Superimposed 2D structure models: Cortisone (**B**), BPH (**D**), BPG (**F**), BPAME (**H**): each inhibitor (purple) having overlapping residues (circled in red) with cortisone (cyan). Dash-line indicates the hydrogen bond.

The crystal structure of human 11β-HSD1 (PDB id: 4C7K) was adopted as the target for docking cortisone. Cortisone forms two hydrogen bonds with catalytic residues Ser170 (3.28Å) and Tyr183 (3.03Å) (Figure 6A,B). First, we analyzed the most potent BPA analog, BPH, and found that BPH forms hydrogen bonds with residues Ser170 and Leu215 and hydrophobic interactions with 13 residues (Figure 6C,D), and ΔG was −8.72 kcal/mol (Table 2). Then, we analyzed the second potent chemical, BPG, and found that BPG forms hydrogen bonds with residues Ser170 and Ala172 and hydrophobic interactions with 12 residues (Figure 6E,F). We analyzed the weaker chemical, BPAME, and found that BPAME forms a hydrogen bond with residue Lys187 and hydrophobic interactions with 12 residues (Figure 6G,H) but does not form a hydrogen bond with residue Ser170. We also found that other BPA analogues, DABPA and DMBPA, bind to the steroid binding cavity (Figure S1). They also form hydrogen bonds with residue Ser170 (Figure S1). We analyzed BPA (positive control) and 4-nonyphenol (4-NP, negative control) and found that BPA forms a hydrogen bond with Ser170, while 4-NP does not form this hydrogen bond (Figure S1).

### 2.4. Molecular Docking Analysis of BPA Analogues with Rat 11β-HSD1

**Figure 7:**
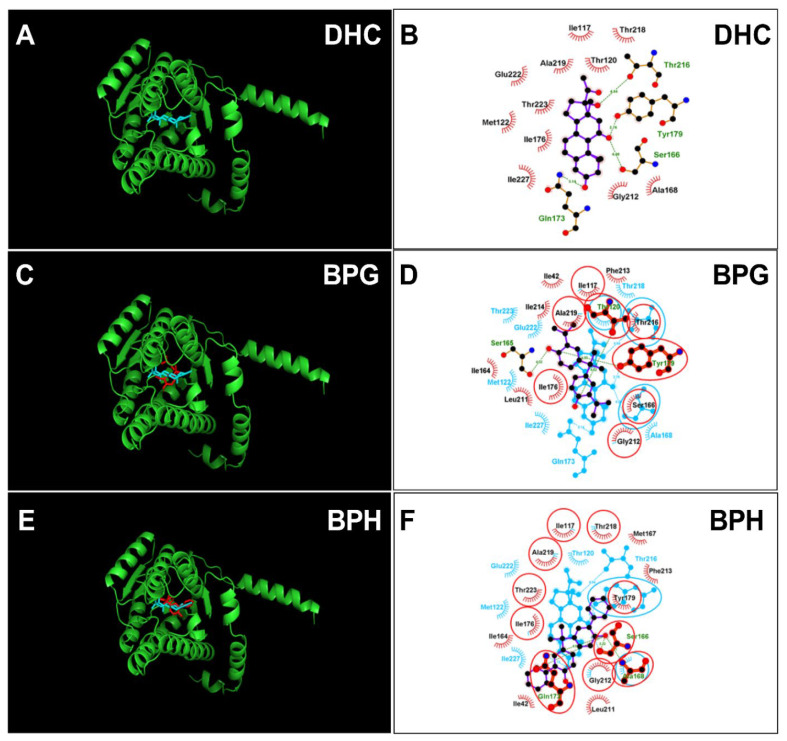
Molecular docking of 11-dehydrocorticosterone (DHC), bisphenol G (BPG), bisphenol H (BPH) with rat 11β-HSD1. Superimposed 3D structure models (BPA analogues = red; cortisone = cyan): DHC (**A**), BPG (**C**), BPH (**E**); Superimposed 2D structure models: DHC (**B**), BPG (**D**), BPH (**F**): each inhibitor (purple) having overlapping residues (circled in red) with cortisone (cyan). Dash-line indicates the hydrogen bond.

The crystal structure of rat 11β-HSD1 was unavailable, and an AlphaFold2-constructed 3D structure (AF-P16232-F1-model_v4) was adopted as the target for molecular docking analysis. The structure was vilified by Ramachandran plot analysis ProCheck software in the UCLA-DOE LAB-SAVES v6.0 (https://saves.mbi.ucla.edu/, (accessed on 11 May 2023)). It was found that 100 percent of residues fell inside the favored (94.6 percent) and the additionally allowed (5.4 percent) regions, whereas no residues fell within the disallowed region for rat 11β-HSD1 (Figure S2A,B), confirming the 3D structure validity of rat 11β-HSD1. DHC was docked with this enzyme, and the 11β-hydroxyl group of DHC forms two hydrogen bonds with catalytic residues Ser166 and Tyr179 and two additional residues (Figure 7A,B), confirming suitable binding of DHC. We analyzed BPG and BPH and found that both bind to the steroid binding site of rat 11β-HSD1 (Figure 7C–F) and that BPG forms hydrogen bonds with residues Thr120, Ser165, and Tyr179 (Figure 7C,D) and BPH forms hydrogen bonds with residues Ser166, Ala168, and Gln173 (Figure 7E,F). ΔG values were −5.64 kcal/mol for BPG and −5.40 kcal/mol for BPH (Table 1). We also analyzed BPAME and DABPA, whose docking analysis is shown in Figure S3. The binding cavity analysis of human and rat 11β-HSD1 revealed that the human enzyme has a larger binding volume (1917.38 Å for human 11β-HSD1 vs. 1476.61 Å for rat one, Figure S4), indicating that rat 11β-HSD1 has a smaller binding cavity than the human enzyme. We analyzed BPA (positive control) and 4-nonyphenol (4-NP, negative control) (Figure S3).

### 2.5. Bivariate Correlation Analysis for Inhibitory Strength BPA Analogues with Structural Features

**Figure 8:**
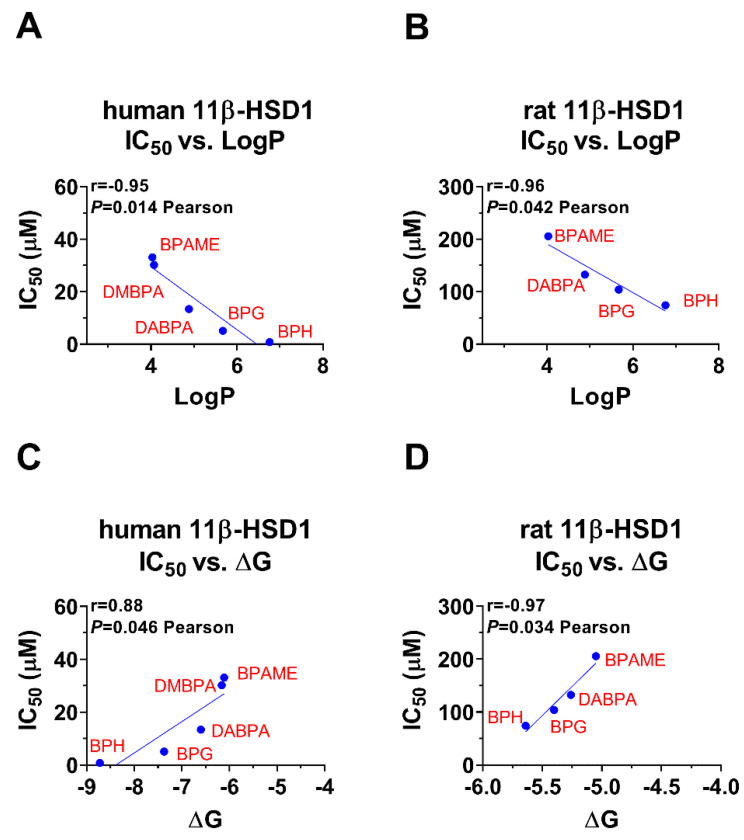
Correlation regression analysis of inhibition constant and LogP and the lowest binding energy (ΔG): (**A**,**C**): The effects of BPA analogues on human 11β-HSD1; (**B**,**D**) the effects of BPA analogues on rat 11β-SD1.

Bivariate correlation analysis showed that LogP was inversely correlated with the IC_50_ values, meaning that the larger the LogP, the lower the IC_50_ value, and thus the more potent inhibition (Figure 8A,B). Bivariate correlation analysis showed that ΔG was positively correlated the IC_50_ values, meaning that the larger the ΔG, the higher the IC_50_ value, and thus the less potent inhibition (Figure 8C,D). The bivariate correlation analysis between each BPA analogue’s structural parameters was listed (Figures S5 and S6).

### 2.6. ADMET Prediction of BPA Analogues

The aqueous solubility level was BPA > BPAME = BPG = DABPA = DMBPA = TMBPA > BPH, and they were found to be able to transport across the BBB. They were found to be inhibitors of CYP2D6, except TMBPA. In addition, BPH and BPG had lower absorption levels in the human intestine, and they all had hepatotoxic potential. They were also found to be highly bound with plasma proteins (Supplementary Table S3 and Figure S7).

## 3. Discussion

BPA is a chemical commonly used in the production of certain plastics and resins. However, BPA has been found to have adverse effects on human health, particularly in terms of endocrine disruption [1,3]. This has led to the development and use of BPA analogues, which are structurally similar compounds with similar properties.

In this study, among these BPA analogues, alkyl and aryl BPA analogues were found to inhibit human and rat 11β-HSD1, an enzyme involved in cortisol/CORT metabolism [16]. However, the degree of inhibition varies depending on the structure of the analog. In particular, it has been observed that LogP, a measure of the compound’s lipophilicity, is inversely correlated with IC_50_, the concentration at which 50% inhibition occurs. This suggests that compounds with higher lipophilicity are more effective inhibitors of 11β-HSD1.

The concept of lipophobicity relates to the fact that many chemicals are either soluble in lipids or not. Since the catalytic domains of human and rat 11β-HSD1 enzymes contain 12–13 hydrophobic residues, in the case of bioactive substances, the more lipophilic a molecule is, the better it will be able to bind to the hydrophobic cavity. In contrast, lipophobic substances will have less ability to do so. With this in mind, it is not surprising that increased lipophobicity within the structure of a molecule can have an inhibitory effect on its activity.

The increased substitute group size on the benzene ring of BPA, specifically from methyl to benzyl, in relation to the inhibitory effect on substances such as DMBPA, DABPA, BPG, and BPH presents an interesting perspective on chemical structure and its impact on behavior. It appears from the study that with a large size substitution on the benzene ring, there is an increased inhibition (BPH > BPG > DABPA > DMBPA) when compared to BPA. This inhibition is possibly due to an increase in lipophobicity. It is true that the benzyl substitutions on the benzene ring, as in BPH, significantly increase the LogP value. Although BPG and DABPA have the same carbon number, the double bond allyl group in DABPA causes the reduction in LogP, thereby leading to a weaker inhibition than BPG. Interestingly, the ether in BPAME significantly increases the LogP (4.04 in BPAME vs. 3.42 in BPA), thereby increasing the inhibitory strength when compared to BPA.

The study also noted that TMBPA was weaker than DMBPA to inhibit human and rat 11β-HSD1 enzymes, despite having an extra methyl group, which would generally increase lipophobicity (LogP: 4.66 in TMBPA vs. 4.04 in DMBPA). The reasoning is that the additional methyl group may increase the space hindrance. This essentially means that the extra group creates more spatial challenges for the molecule to interact with its target, which could result in less activity.

In this study, unlike human 11β-HSD1, we found that BPA analogues are much weaker in their ability to inhibit rat 11β-HSD1 when compared to BPA. For example, when the IC50 values are compared, BPH is about 99 times more potent to inhibit human 11β-HSD1 than rat 11β-HSD1, and BPG is about 21 times more potent to inhibit the human enzyme than the rat enzyme (Table 2). Although the exact mechanism of the species difference remains unclear, previous studies have identified a species-dependent difference for 11β-HSD1 inhibitors [21]. This could be the substrate affinity difference. CORT is the major glucocorticoid in rats, whereas cortisol is the major glucocorticoid in humans, thereby limiting the use of these most commonly used rodents to study effects on glucocorticoid metabolism. Our docking analysis for the steroid binding pocket for 11β-HSD1 enzymes also shows that human 11β-HSD1 with cortisol has a larger binding cavity than the rat isoform (Fig.S4). This could explain why the increased substitution group size on the benzene ring (isopropyl group for BPG and benzyl group for BPH) cannot well fit the smaller binding cavity of rat 11β-HSD1.

The human and rat 11β-HSD1 enzymes belong to the SDR family, which has catalytic residues Ser…Tyr-X-X-X-Lys [16]. Docking analysis showed that all BPA analogues bind to the steroid catalytic cavity, mostly contacting these catalytic residues, indicating that they are competitive or mixed/competitive inhibitors. Ser residue in the catalytic triad domain Ser…Tyr-X-X-X-Lys has been indicated to play a critical role in the proton transfer between cortisone and NADPH, and the mutation of Ser170 inactivates 11β-HSD1 [22]. The binding to Ser residue in human 11β-HSD1 by BPG and BPH indicates that BPG and BPH are more potent than four other BPA analogues to inhibit human 11β-HSD1, since four other BPA analogues do not bind to Ser170 residue.

Although the crystal structure of human 11β-HSD1 is available, the structure of rat 11B-HSD1 remains unavailable. This presents a challenge for studying the effects of BPA analogues on this enzyme in rats. However, after analyzing the differences and similarities between rat and human 11β-HSD1, we noted a subtle difference between the two: the binding pocket size of human 11β-HSD1 is larger than that of the rat enzyme (Figure S4). It remains unclear whether this difference contributes to the distinct species difference that has been observed.

In addition, it has been found that binding energy, or the energy required for the compound to form a stable complex with the enzyme, is positively correlated with IC_50_. This suggests that compounds with stronger binding affinity to 11β-HSD1 are also more effective inhibitors.

It is important to note that the use of BPA analogues does not necessarily mean that they are a safe alternative to BPA. This study found that the inhibitory strength of BPA analogues was BPH > BPG > DABPA > DMBPA > BPAME > TMBPA = BPA, indicating that they are more potent to induce dysfunction due to cortisol deficiency. However, only BPH has a sub-micromolar IC_50_ value, while others are much weaker, indicating that other BPA analogues except BPH might be safe. It is emphasized that care should be taken when extrapolating results obtained from the rat model to humans.

The inhibition of 11β-HSD1 may lead to adverse effects. Cortisol is an important hormone involved in the body’s stress response, and inhibition of 11β-HSD1 can result in cortisol deficiency. This can lead to adverse effects such as fetal lung development disorders, excessive ACTH stimulation, and liver fibrosis.

BPA analogues also target other steroidogenic enzymes. For example, BPA or BPA analogues can inhibit human and rat 11β-HSD2 (another 11β-HSD1 isoform with the opposite catalytic reaction) [23,24], human placental 3β-HSD1 (an enzyme for the conversion of pregnenolone to progesterone) [25], human and rat CYP17A1 and aromatase activity [25,26].

We also predicted that the aqueous solubility level was BPA > BPAME = BPG = DABPA = DMBPA= TMBPA > BPH, and they were found to be able to transport across the BBB and inhibit the drug metabolic enzyme CYP2D6, except TMBPA. In addition, BPH and BPG had lower absorption levels in the human intestine and all had hepatotoxic potential. These results suggest that these chemicals might target the tissues to inhibit 11β-HSD1.

## 4. Materials and Methods

### 4.1. Materials and Chemicals

11-dehydrocorticosterone (DHC), corticosterone (CORT), cortisone, and cortisol were obtained from Steraloids (Newport, RI, USA), BPA (CAS#: 80-05-7, CAT# 239658, purity 99%), dimethyl sulfoxide (DMSO), G6P, and NADPH from Sigma-Aldrich (St. Louis, MO, USA), 4,4′-(propane-2,2-diyl)bis(2-isopropylphenol) (BPG, CAS# 127-54-8, CAT# BD297061, purity 95%), 4,4′-(propane-2,2-diyl)bis(methoxybenzene) (BPAME, CAS# 1568-83-8, CAT# R118631, purity 95%) and 2,2′-diallyl BPA (DABPA, CAS# 1745-89-7, CAT# R039218, purity 85%) from Rhawn (Shanghai, China), 2,2-bis(2-hydroxy-5-biphenylyl)propane (BPH, CAS# 24038-68-4, CAT# B2750, purity 98%) and 2,2-bis(4-hydroxy-3-methylphenyl)propane (DMBPA, CAS# 79-97-0, CAT# B1567, purity > 98%) from TCI (Shanghai, China), and 2,2-bis(4-hydroxy-3,5-dimethylphenyl)propane (TMBPA, CAS# 5613-46-7, CAT# 409830, purity 98%) from JK Chemical (Beijing, China). The human 11β-HSD1 enzyme in the human liver microsome (CAT# H2610) was obtained from Iphase (Beijing, China). The rat 11β-HSD1 enzyme in the rat liver microsome was prepared from livers of male Sprague-Dawley rats (age 3 months) from Shanghai Laboratory Animal Center (Shanghai, China) under the approval of the Wenzhou Medical University Institutional Animal Care and Use Committee (wydw2020-0801).

### 4.2. Preparation of Rat Liver Microsomes

The preparation of rat liver microsomes was carried out as previously reported [17]. In this preparation procedure, rat livers were homogenized in sucrose (0.25 M) containing PBS (0.01 M, pH 7.2). The homogenized samples were sequentially centrifuged at 700× *g* for 30 min, at 14,500× *g* for 30 min, and at 105,000× *g* for 60 min twice to produce the microsomal pellet. The pellets were resuspended in cold PBS by homogenization, and the concentration of microsomal protein was determined using the Bio-Rad Protein Assay Kit (CAT#: P0010; Beyotime, Jiangsu, China) according to the manufacturer’s instructions.

### 4.3. 11β-HSD1 Assay

The 11β-HSD1 activity was determined by measuring the reductive catalysis of 11keto-steroid substrates, cortisone for human 11β-HSD1 and DHC for rat 11β-HSD1, which were converted to cortisol and CORT, respectively. The 11β-HSD1 assay was carried out as previously described with a minor modification [17]. Briefly, a 100 µL reaction mixture containing cortisone or DHC, NADPH (0.2 mM)/G6P (1 mM), an enzyme source (1 µg human liver or 5 µg rat liver microsome) or a BPA analog was reacted in a shaking water bath (75 rpm) at 37 °C. The following steps were designed: (1) Determination of time course of 11β-HSD1 reaction: 200 nM cortisone or DHC in the assay system for 0, 15, 30, 60, 90, and 120 min; (2) Determination of Michaelis-Menten kinetics: 0, 0.25, 0.5, 1, 2, 3, and 4 µM cortisone or DHC in the assay system for 15 min; (3) Screening assay: 200 nM cortisone or DHC and 100 µM BPA analog in the system for 15 min; (4) Half-maximum inhibitory concentration (IC_50_) assay: 200 nM cortisone or DHC and 0.1, 1, 5, 10, 25, 50, or 100 µM BPA analogues in the system for 15 min; (5) Mode of action assay: 0, 0.25, 0.5, 1, 2, 3, and 4 µM cortisone or DHC, and 0–50 µM BPA analogues in the system for 15 min. At the end of the reaction, acetonitrile (200 µL, CAT#: UN1648; Sigma) and internal standard testosterone-d5 (IS, T-d5, 10 μL, Zzbio, Shanghai, China) were added to the system, the organic layer was extracted, and 10 µL was injected into the HPLC-MS/MS system to measure cortisol, or CORT, as previously described [27].

### 4.4. Determination of Cortisol and CORT by HPLC-MS/MS Method

The amount of cortisol, or CORT, in the organic layer was determined using an Acquity UPLC equipped with a BEH C18 column (2.1 mm, 50 mm, 1.7 m) and a 0.2 mm stainless steel frit filter and an XEVO TQD triple quadrupole mass spectrometer (Waters, Milford, MA, USA), as previously described [28]. The mobile phase was a mixture of solvents A (0.1% formic acid in water) and B (acetonitrile) combined in the following gradient program: 5–5% B (0–0.5 min), 5–95% B (0.5–1.0 min), 95–95% B (1.0–2.0 min), and 95–5% B (2.0–2.1 min). The injection volume was 6 µL, and the flow rate was 0.40 mL/min. Before the second injection, a re-equilibration period of 0.9 min was set. The temperatures were 4 °C for the sample and 40 °C for column. Mass spectrometric detection was carried out using a Waters Corporation XEVO TQD triple quadruple mass spectrometer with an ESI source. For cortisol and IS, the multiple reaction monitoring mode transitions were *m*/*z* 358.3→340.3 and *m*/*z* 237.2→194.3. Data collection and instrument control were performed using Masslynx 4.1 software (Waters Corp., Milford, MA, USA). Cortisol, or CORT, standards were prepared by dissolving them in methanol. The intra- and inter-assay coefficients of variation for cortisol or CORT were less than 10%. The enzymatic activity obtained by measuring the conversion rate (%) of cortisone to cortisol for human 11β-HSD1 or DHC to CORT for rat 11β-HSD1 was calculated.

### 4.5. Analysis of 11β-HSD1 Kinetics and Inhibitory Parameters of BPA Analogues

The time curve of the 11β-HSD1 reaction was prepared by a linear regression using GraphPad version 8.0 (GraphPad, San Diego, CA, USA). A nonlinear regression fit (curve fit) using the Michaelis-Menten equation (V = Vmax ∗ [substrate]/(Km + [substrate]), where V is 11β-HSD1velocity (pmol/mg/min), Vmax is maximal velocity, [substrate] is substrate concentration, and Km is the Michaelis-Menten constant) was performed by GraphPad, and Km and Vmax were calculated. Inhibitory effects of BPA derivatives at 100 μM were compared to the control (DMSO, setting for 100% residual activity). Dose-response was performed using a range of concentrations of BPA derivatives, and a nonlinear regression (curve fit) of Log[BPA analog] vs. response (three parameters) was carried out by GraphPad to calculate the half-maximal inhibitory concentration (IC_50_). A nonlinear regression in the enzyme kinetics–inhibition (mixed model) to judge the mode of action of the BPA derivative was carried out by GraphPad using the following equation: V = VmaxApp ∗ [substrate]/(KmApp + [substrate]), where VmaxApp is the adjusted Vmax and KmApp is the adjusted Km, VmaxApp = Vmax/(1 + [BPA derivative]/(α ∗ Ki), where [BPA derivative] is the inhibitor concentration, and KmApp = Km ∗ (1 + [BPA derivative]/Ki)/(1 + [BPA derivative]/(α ∗ Ki)), where Ki is the inhibition constant. “α” was used to judge the mode of action: when α is very large, the BPA derivative is a competitive inhibitor; when α is very small but greater than zero, the BPA derivative is an uncompetitive inhibitor; when α is 1, the BPA derivative is a noncompetitive inhibitor; when α > 1 or α < 1, the BPA derivative is a mixed inhibitor. The lineweaver-Burk plot after linear regression was also carried out to judge the mode of action.

### 4.6. Molecular Docking Analysis of Human and Rat 11β-HSD1

The crystal structure of human 11β-HSD1 (PDB id:4C7K) with a synthetic inhibitor and NADP^+^ as selected docking targets [29]. AlphaFold2-generated structure of rat 11β-HSD1 (AF-P16232-F1-model_v4, credibility of 94.7%) based on murine 11β-HSD1 crystal structure [30] was selected for the rat target as described [31,32]. The protein structures of human 11β-HSD1 and rat 11β-HSD1 were optimized as previously described [33,34]. ProCheck software was used to verify the anticipated 3D structures by creating the Ramachandran plot [34]. Cortisone, DHC, and BPA analogues were drawn using ChemBioDraw Ultra 12.0 (Cambridge, UK). The cortisone-binding cavity of human 11β-HSD1 and the DHC-binding cavity of rat 11β-HSD1 were predicted using Proteinplus Server [35]. Docking simulation analysis was carried out by Autodock 4.0 as described [36], and conformation of a chemical with the lowest binding energy (ΔG as kcal/mol) was calculated. Superimposed 3D structure was illustrated by PyMOL software v2.3.2, and superimposed 2D structure was illustrated by LigPlot as described [37]. After docking with human 11β-HSD1, the 11β-hydroxy group of cortisol forms hydrogen bonds with key catalytic residues Ser170 and Tyr183 of human 11β-HSD1, which agrees with a previous reported result [38]. After docking with rat 11β-HSD1, the 11β-hydroxy group of DHC forms key catalytic residues Ser166 and Tyr179 of rat 11β-HSD1, supporting a reliable structure conformation with murine 11β-HSD1 structure [30].

### 4.7. Bivariate Correlation Analysis for Structural Features of BPA Analogues with IC_50_ Values

Structural features, including hydrogen bond donor, hydrogen bond acceptor, heavy atoms, hetero atoms, LogP, fraction sp^3^, ring number, and molecular weight (MW), were obtained from the ZINC database [39]. ΔG was obtained from the docking analysis above. The bivariate correlation between LogP, MW, and ΔG and IC_50_ values of each BPA analogue was analyzed by Pearson correlation analysis.

### 4.8. Drug Metabolism and Pharmacokinetics Prediction

In this study, we used Discovery Studio software v19.1.0 (Omaha, NE, USA) to predict the drug metabolism and pharmacokinetics (ADMET) parameters of seven bisphenols. Physical descriptors such as aqueous solubility, blood–brain barrier (BBB) penetration, human intestinal absorption (HIA), hepatotoxicity, cytochrome P450 2D6 (CYP2D6) inhibition, and plasma protein binding (PPB) were generated from the analysis (Supplementary Table S3).

### 4.9. Statistics

The experiments were repeated four to six times. The inhibitory potency of BPA analogues at 100 µM was compared by a one-way ANOVA, followed by a post hoc Dunnett’s multiple comparisons test to judge any significant difference between BPA analogues and the control. The significant difference in IC_50_ values between each BPA analog was judged by one-way ANOVA followed by a post hoc Turkey’s multiple comparisons test. Data were presented as means ± SEM, and differences were statistically significant at * *p* < 0.05, ** *p* < 0.01, and *** *p* < 0.001.

## 5. Conclusions

In conclusion, in this study, we found that 11β-HSD1 is a new potential target of BPA analogues. BPA analogues with substitutions on the benzene ring significantly increase the binding to human 11β-HSD1, thereby leading to more potent inhibition of the enzyme activity. The inhibitory strength of BPA analogues was BPH > BPG > DABPA > DMBPA > BPAME > TMBPA = BPA, indicating that they are more potent to induce dysfunction due to cortisol deficiency. However, there is species-dependent inhibition with the rat enzyme, which is insensitive to BPA analogues. Hydrophobicity seems to determine the inhibitory strength of BPA analogues on both enzymes. Docking analysis reveals the binding potency of BPA analogues to 11β-HSD1 might depend on lipophilicity, molecular weight, molecular volume, and heavy atoms.

## Figures and Tables

**Table 1:**
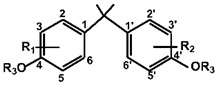
Chemical structures of bisphenols.

Compound	R1(C3)	R2(C3′)	R3(C4,4′ -O-)	
BPA	H	H	H	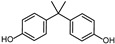
BPG	3-CH2(CH3)2	3′-CH2(CH3)2	H	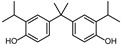
BPH	3-C6H5	3′-C6H5	H	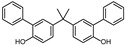
BPAME	H	H	CH3	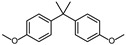
DABPA	3-CH2CH2CH2	3**′**-CH2CH2CH2	H	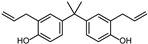
DMBPA	3-CH3	3**′**-CH3	H	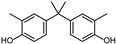
TMBPA	3-CH3,5-CH3	3**′**-CH3,5**′**-CH3	H	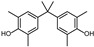

**Table 2:** Inhibitory potencies on human and rat 11β-HSD1 and docking parameters of bisphenols.

Compound	IC_50_ μM	Ki μM	Cal. Ki μM	LBE (kcal/mol)	Mode Action	Binding Site
Human 11β-HSD1 (PDB id: 4C7K)						
BPA	297.4 ± 2.99	ND	ND	ND	ND	ND
BPAME	33.08 ± 0.57	33.81	33.34	−6.11	Competitive	Steroid
BPG	5.06 ± 0.07	5.33	3.96	−7.37	Competitive	Steroid
BPH	0.75 ± 0.02	0.44	0.40	−8.72	Competitive	Steroid
DABPA	13.36 ± 0.19	14.04	14.52	−6.60	Competitive	Steroid
DMBPA	30.18 ± 0.71	30.97	30.81	−6.16	Competitive	Steroid
TMBPA	319.16 ± 2.78	ND	ND	ND	ND	ND
Rat 11β-HSD1 (AlphaFold id: AF-P16232-F1-model_v4)						
BPA	36.76 ± 1.09	ND				
BPAME	205.74 ± 2.16	ND	204.2	−5.05	ND	Steroid
BPG	103.92 ± 1.52	88.65	106.97	−5.40	Mixed	Steroid
BPH	74.22 ± 0.98	74.56	73.73	−5.64	Competitive	Steroid
DABPA	132.4 ± 1.66	ND	133.51	−5.26	ND	Steroid

IC_50_ = half maximal inhibitory concentration; Ki = inhibition constant (measured); Cal. Ki = inhibition constant (calculated); LBE = lowest binding energy; Mean ± SEM, *n* = 4.

## Data Availability

Data will be made available on request.

## Supplementary Materials

The following supporting information can be downloaded at: https://www.mdpi.com/article/10.3390/molecules28134894/s1. Table S1. Information of bisphenol A and its analogues. Table S2: ZINC Information of bisphenol A and its analogues. Table S3: Drug metabolism and pharmacokinetics of bisphenol A analogues. Figure S1. Molecular docking of bisphenol A analogs with human 11β-HSD1. Figure S2. The Ramachandran figure illustrates the phi-psi torsion angles for human 11β-HSD1 (A) and rat 11β-HSD1 (B). Figure S3. Molecular docking of bisphenol A analogs with rat 11β-HSD1. Figure S4. Binding cavity of human and rat 11β-HSD1 enzymes. Figure S5. Bivariate correlation analysis for structural features of BPA analogues with IC50 values on human 11β-HSD1 enzyme. Figure S6. Bivariate correlation analysis for structural features of BPA analogues with IC50 values on rat 11β-HSD1 enzyme. Figure S7. ADMET prediction of BPA analogues.

## Sample Availability

Samples of the compounds are available from the authors.

## References

[1] Li X., Wen Z., Wang Y., Mo J., Zhong Y., Ge R.S. (2020). Bisphenols and Leydig Cell Development and Function. Front. Endocrinol..

[2] Patisaul H.B. (2020). Achieving CLARITY on bisphenol A, brain and behaviour. J. Neuroendocrinol..

[3] Kim M.J., Park Y.J. (2019). Bisphenols and Thyroid Hormone. Endocrinol. Metab..

[4] Kim J.J., Kumar S., Kumar V., Lee Y.M., Kim Y.S., Kumar V. (2019). Bisphenols as a Legacy Pollutant, and Their Effects on Organ Vulnerability. Int. J. Environ. Res. Public Health.

[5] Santoro A., Chianese R., Troisi J., Richards S., Nori S.L., Fasano S., Guida M., Plunk E., Viggiano A., Pierantoni R. (2019). Neuro-toxic and Reproductive Effects of BPA. Curr. Neuropharmacol..

[6] Ma Y., Liu H., Wu J., Yuan L., Wang Y., Du X., Wang R., Marwa P.W., Petlulu P., Chen X. (2019). The adverse health effects of bisphenol A and related toxicity mechanisms. Environ. Res..

[7] Dallio M., Diano N., Masarone M., Gravina A.G., Patane V., Romeo M., Di Sarno R., Errico S., Nicolucci C., Abenavoli L. (2019). Chemical Effect of Bisphenol A on Non-Alcoholic Fatty Liver Disease. Int. J. Environ. Res. Public Health.

[8] Cesen M., Lenarcic K., Mislej V., Levstek M., Kovacic A., Cimrmancic B., Uranjek N., Kosjek T., Heath D., Dolenc M.S. (2018). The occurrence and source identification of bisphenol compounds in wastewaters. Sci. Total Environ..

[9] Shi L., Li J., Tian F., Tang Y., Wang S., Li Q., Zhu Y., Zhu Q., Ge R.S. (2022). Dimethylbisphenol A inhibits the differentiation of stem Leydig cells in adult male rats by androgen receptor (NR3C4) antagonism. Toxicol. Lett..

[10] Xue J., Kannan K. (2019). Mass flows and removal of eight bisphenol analogs, bisphenol A diglycidyl ether and its derivatives in two wastewater treatment plants in New York State, USA. Sci. Total Environ..

[11] Grimaldi M., Boulahtouf A., Toporova L., Balaguer P. (2019). Functional profiling of bisphenols for nuclear receptors. Toxicology.

[12] Zhang H.C., Hu X.L., Yin D.Q., Lin Z.F. (2011). Development of molecular docking-based binding energy to predict the joint effect of BPA and its analogs. Hum. Exp. Toxicol..

[13] Champagne F.A. (2013). Early environments, glucocorticoid receptors, and behavioral epigenetics. Behav. Neurosci..

[14] Ohshima M., Ohno S., Nakajin S. (2005). Inhibitory effects of some possible endocrine-disrupting chemicals on the isozymes of human 11beta-hydroxysteroid dehydrogenase and expression of their mRNA in gonads and adrenal glands. Environ. Sci..

[15] Zhou H.Y., Chen X.X., Lin H., Fei A.L., Ge R.S. (2014). 11beta-hydroxysteroid dehydrogenase types 1 and 2 in postnatal development of rat testis: Gene expression, localization and regulation by luteinizing hormone and androgens. Asian J. Androl..

[16] Chapman K., Holmes M., Seckl J. (2013). 11beta-hydroxysteroid dehydrogenases: Intracellular gate-keepers of tissue glucocorticoid action. Physiol. Rev..

[17] Guo J., Yuan X., Qiu L., Zhu W., Wang C., Hu G., Chu Y., Ye L., Xu Y., Ge R.S. (2012). Inhibition of human and rat 11beta-hydroxysteroid dehydrogenases activities by bisphenol A. Toxicol. Lett..

[18] Hewitt K.N., Walker E.A., Stewart P.M. (2005). Minireview: Hexose-6-phosphate dehydrogenase and redox control of 11{beta}-hydroxysteroid dehydrogenase type 1 activity. Endocrinology.

[19] Tannin G.M., Agarwal A.K., Monder C., New M.I., White P.C. (1991). The human gene for 11 beta-hydroxysteroid dehydrogenase. Structure, tissue distribution, and chromosomal localization. J. Biol. Chem..

[20] Ge R.S., Gao H.B., Nacharaju V.L., Gunsalus G.L., Hardy M.P. (1997). Identification of a kinetically distinct activity of 11beta-hydroxysteroid dehydrogenase in rat Leydig cells. Endocrinology.

[21] Arampatzis S., Kadereit B., Schuster D., Balazs Z., Schweizer R.A., Frey F.J., Langer T., Odermatt A. (2005). Comparative enzymology of 11beta-hydroxysteroid dehydrogenase type 1 from six species. J. Mol. Endocrinol..

[22] Obeid J., White P.C. (1992). Tyr-179 and Lys-183 are essential for enzymatic activity of 11 beta-hydroxysteroid dehydrogenase. Biochem. Biophys. Res. Commun..

[23] Shi L., Zhang B., Ying Y., Tang Y., Wang S., Zhu Y., Li H., Ge R.S., Liu Y. (2023). Halogen atoms determine the inhibitory potency of halogenated bisphenol A derivatives on human and rat placental 11beta-hydroxysteroid dehydrogenase 2. Food Chem. Toxicol. Int. J. Publ. Br. Ind. Biol. Res. Assoc..

[24] Zhang B., Wang S., Tang Y., Hu Z., Shi L., Lu J., Li H., Wang Y., Zhu Y., Lin H. (2023). Direct inhibition of bisphenols on human and rat 11beta-hydroxysteroid dehydrogenase 2: Structure-activity relationship and docking analysis. Ecotoxicol. Environ. Saf..

[25] Tang L., Shi L., Tang Y., Ying Y., Dong Y., Li H., Ge R.S. (2022). Chemicals of environmental concern as inhibitors of human placental 3beta-hydroxysteroid dehydrogenase 1 and aromatase: Screening and docking analysis. Chem. Biol. Interact..

[26] Ye L., Zhao B., Hu G., Chu Y., Ge R.S. (2011). Inhibition of human and rat testicular steroidogenic enzyme activities by bisphenol A. Toxicol. Lett..

[27] Wang S., Zhang B., Zhai Y., Tang Y., Lou Y., Zhu Y., Wang Y., Ge R.S., Li H. (2022). Structure-activity relationship analysis of perfluoroalkyl carbonic acids on human and rat placental 3beta-hydroxysteroid dehydrogenase activity. Toxicology.

[28] Zhao X., Ji M., Wen X., Chen D., Huang F., Guan X., Tian J., Xie J., Shao J., Wang J. (2021). Effects of Midazolam on the Development of Adult Leydig Cells from Stem Cells In Vitro. Front. Endocrinol..

[29] Goldberg F.W., Dossetter A.G., Scott J.S., Robb G.R., Boyd S., Groombridge S.D., Kemmitt P.D., Sjogren T., Gutierrez P.M., de Schoolmeester J. (2014). Optimization of brain penetrant 11beta-hydroxysteroid dehydrogenase type I inhibitors and in vivo testing in diet-induced obese mice. J. Med. Chem..

[30] Zhang J., Osslund T.D., Plant M.H., Clogston C.L., Nybo R.E., Xiong F., Delaney J.M., Jordan S.R. (2005). Crystal structure of murine 11 beta-hydroxysteroid dehydrogenase 1: An important therapeutic target for diabetes. Biochemistry.

[31] Jumper J., Evans R., Pritzel A., Green T., Figurnov M., Ronneberger O., Tunyasuvunakool K., Bates R., Žídek A., Potapenko A. (2021). Highly accurate protein structure prediction with AlphaFold. Nature.

[32] Varadi M., Anyango S., Deshpande M., Nair S., Natassia C., Yordanova G., Yuan D., Stroe O., Wood G., Laydon A. (2022). AlphaFold protein structure database: Massively expanding the structural coverage of protein-sequence space with high-accuracy models. Nucleic Acids Res..

[33] Ongtanasup T., Wanmasae S., Srisang S., Manaspon C., Net-Anong S., Eawsakul K. (2022). In silico investigation of ACE2 and the main protease of SARS-CoV-2 with phytochemicals from Myristica fragrans (Houtt.) for the discovery of a novel COVID-19 drug. Saudi J. Biol. Sci..

[34] Ongtanasup T., Mazumder A., Dwivedi A., Eawsakul K. (2022). Homology Modeling, Molecular Docking, Molecular Dynamic Simulation, and Drug-Likeness of the Modified Alpha-Mangostin against the beta-Tubulin Protein of Acanthamoeba Keratitis. Molecules.

[35] Schoning-Stierand K., Diedrich K., Ehrt C., Flachsenberg F., Graef J., Sieg J., Penner P., Poppinga M., Ungethum A., Rarey M. (2022). ProteinsPlus: A comprehensive collection of web-based molecular modeling tools. Nucleic Acids Res..

[36] Thomas J.L., Mason J.I., Brandt S., Spencer B.R., Norris W. (2002). Structure/function relationships responsible for the kinetic differences between human type 1 and type 2 3beta-hydroxysteroid dehydrogenase and for the catalysis of the type 1 activity. J. Biol. Chem..

[37] Wallace A.C., Laskowski R.A., Thornton J.M. (1995). LIGPLOT: A program to generate schematic diagrams of protein-ligand interactions. Protein Eng..

[38] Goldberg F.W., Leach A.G., Scott J.S., Snelson W.L., Groombridge S.D., Donald C.S., Bennett S.N., Bodin C., Gutierrez P.M., Gyte A.C. (2012). Free-Wilson and structural approaches to co-optimizing human and rodent isoform potency for 11beta-hydroxysteroid dehydrogenase type 1 (11beta-HSD1) inhibitors. J. Med. Chem..

[39] Irwin J.J., Shoichet B.K. (2005). ZINC—A free database of commercially available compounds for virtual screening. J. Chem. Inf. Model..

