# Supplementary material for: Bisphenol A Analogues Inhibit Human and Rat 11β-Hydroxysteroid Dehydrogenase 1 Depending on Its Lipophilicity
Source: Molecules. 2023 Jun 21;28(13):4894. doi: 10.3390/molecules28134894 (PMC10343216; doi:10.3390/molecules28134894)
Supplement: Supplementary file 1 [file molecules-28-04894-s001.zip › molecules-2421234-supplementary.pdf]

Supplementary Materials

Bisphenol A analogues inhibit human and rat 11β-hydroxysteroid dehydrogenase 1 depending on its lipophilicity

Hong Wang [1,2,3,4], Jianmin Sang [1,2,3], Zhongyao Ji [1,2,3], Yang Yu [1,2,3], Shaowei Wang [5], Yang Zhu [1,2,3], Huitao Li [1,2,3], Yiyan Wang [1,2,3], Qiqi Zhu[1,2,3,*], Renshan Ge [1,2,3,4,*]

1 Department of Anesthesiology and Perioperative Medicine, The Second Affiliated Hospital and Yuying Chil-dren's Hospital of Wenzhou Medical University, Wenzhou 325027, China

2 Key Laboratory of Pediatric Anesthesiology, Ministry of Education, Wenzhou 325027, China

3 Key Laboratory of Anesthesiology of Zhejiang Province, Wenzhou Medical University, Wenzhou 325027, China

4 Key Laboratory of Structural Malformations in Children of Zhejiang Province and Key Laboratory of Male Health and Environment of Wenzhou, Wenzhou 325000, China

5 Department of Obstetrics and Gynecology, The Second Affiliated Hospital and Yuying Children's Hospital of Wenzhou Medical University, Wenzhou 325027, China

* Correspondence: zhuqiqiwz@wmu.edu.cn (Q.Z.); renshan_ge@wmu.edu.cn (R.G.)

Table S1. Information of bisphenol A and its analogues：ID, name, CAS number (CAS no), catalogue number (CAT#), purity, and company (city, state/region).

ID	Name	CAS no	CAT#	Purity (%)	Company (City, State)	Regulation
BPA	4,4′-Isopropylidenebiphenol	80-05-7	239658	99%	Sigma-Aldrich (St. Louis, MO)	Regulation 10/2011/EU (Ban: baby bottle); The United Nations Environment Program (UNEP): bisphenols as a priority substance for global action. Stockholm Convention on Persistent Organic Pollutants, which aims to eliminate or restrict the production and use of some bisphenols.
BPAME	4,4'-(Propane-2,2-diyl) bis (methoxy benzene)	1568-83-8	R118631	95%	Rhawn (Shanghai, China)	
BPG	4,4'-(Propane-2,2-diyl bis(2-isopropylphenol)	127-54-8	BD297061	95%	Rhawn (Shanghai, China)	
BPH	2,2-Bis(2-hydroxy-5-biphenylyl)propane	24038-68-4	B2750	98%	TCI (Shanghai, China)	
DABPA	2,2'-Diallyl bisphenol A	1745-89-7	R039218	85%	Rhawn (Shanghai, China)	
DMBPA	2,2-Bis(4-hydroxy-3-methylphenyl)propane	79-97-0	B1567	>98%	TCI (Shanghai, China)	
TMBPA	2,2-Bis(4-hydroxy-3,5-dimethylphenyl)propane	5613-46-7	409830	98%	JK Chemical (Beijing, China)	

Table S2: ZINC Information of bisphenol A and its analogues: ID, molecular weight (Mwt), LogP, ring number, heavy atoms, hetero atoms, fraction sp^3, net charge, H-bond donors, H-bond acceptors, tPSA Å², rotatable bonds, apolar desolvation (kcal/mol), polar desolvation (kcal/mol), Molecular volume (Å^3), length (Å), gibbers energy.

ID	BPA	BPAME	BPG	BPH	DABPA	DMBPA	TMBPA
**Molecular weight (Mwt)**	228.291	256.345	312.453	380.487	308.421	256.345	284.399
**LogP**	3.424	4.03	5.671	6.758	4.881	4.041	4.657
**ring number**	2	2	2	4	2	2	2
**heavy Atoms**	17	19	23	29	23	19	21
**hetero Atoms**	2	2	2	2	2	2	2
**fraction sp^3**	0.2	0.29	0.43	0.11	0.24	0.29	0.37
**net charge**	0	0	0	0	0	0	0
**H-bond donors**	2	0	2	2	2	2	2
**H-bond acceptors**	2	2	2	2	2	2	2
**tPSA Å²**	40	18	40	40	40	40	40
**rotatable bonds**	2	4	4	4	6	2	2
**apolar desolvation (kcal/mol)**	3.86	8.17	7.92	11.38	7.95	5.35	6.84
**polar desolvation (kcal/mol)**	-6.23	-5.68	-5.32	-7.24	-6.11	-6.2	-6.03
**molecular (Å^3)**	236.83	279.42	347.68	389.8	363.13	279.28	317.63
**length(Å)**	10.1	12.7	9.9	10.69	9.9	10.2	11.1
**gibbs energy**	-6.16	90.66	20.22	300.44	-96.3	-8.58	-11

Notes:

Molecular Volume (Molvol) was analyzed by MolEdit (C) 2023 Molsoft (San Diego, CA). The length of molecules was calculated by Chemdraw 3D v17.1.0.105.

Table S3: Drug metabolism and pharmacokinetics of bisphenol A analogues.

ID	Solubility level	BBB level	CYP2D6	Hepatotoxicity	HIA	PPB level
BPA	3	1	1	1	0	1
BPAME	2	0	1	1	0	1
BPG	2	0	1	1	1	1
BPH	1	4	1	1	2	1
DABPA	2	0	1	1	0	1
DMBPA	2	1	1	1	0	1
TMBPA	2	0	0	1	0	1

Notes:

Solubility Level: 1(very low), 2(low), 3(good); BBB level (Blood Brain Barrier level): 0 (very high), 1(high), 2 (medium), 3 (low), 4 (undefined); CYP2D6 (c Cytochrome P450 2D6 level): 0 (non-inhibitor), 1(inhibitor); Hepatotoxicity: 0 (non-toxic), 1 (toxic); HIA (Human intestinal absorption level): 0 (good absorption), 1 (moderate absorption), 2 (low absorption); PPB level (Plasma Protein Binding): 0 (binding<90%), 1 (binding>90%).

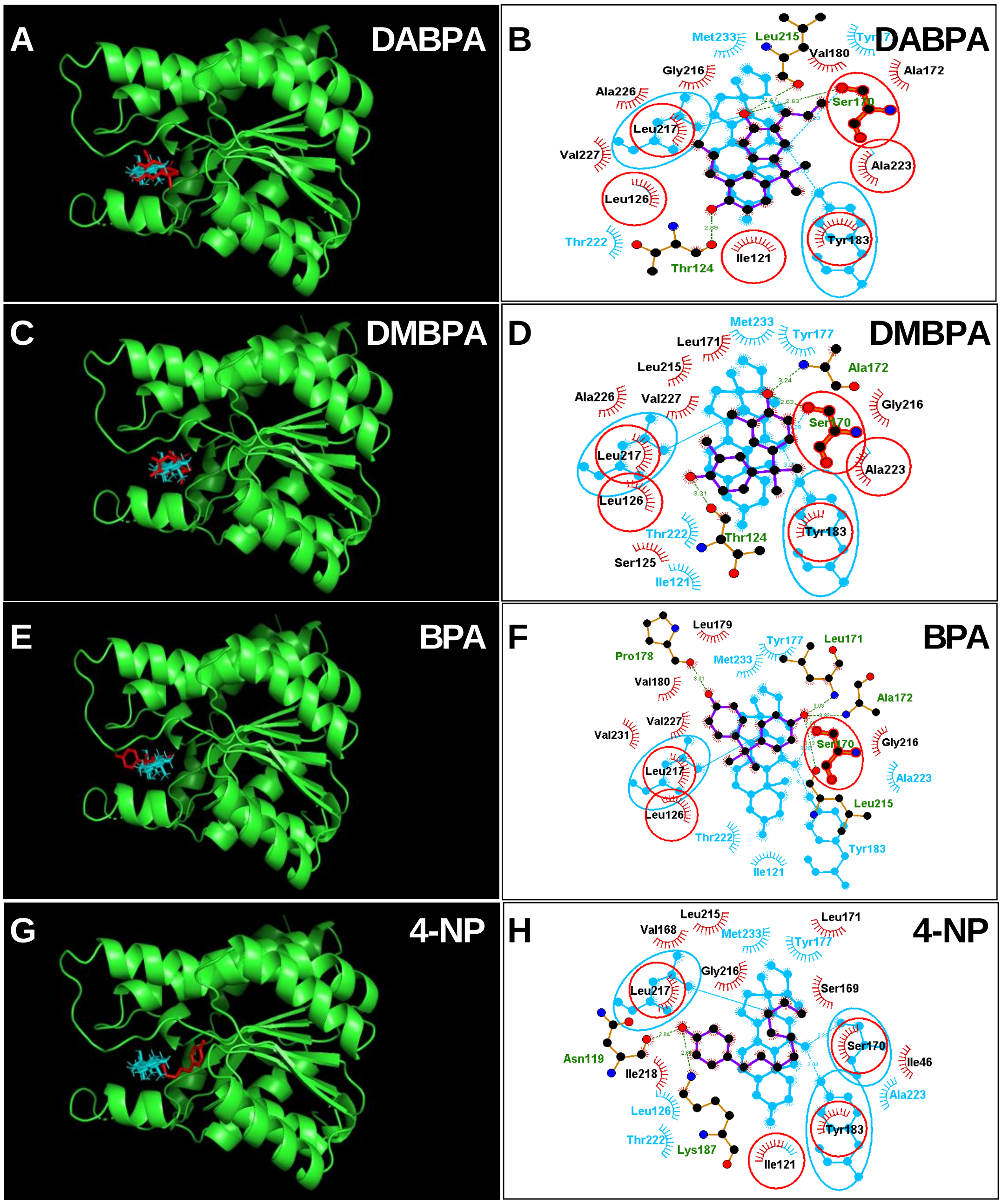

Figure S1. Molecular docking of bisphenol A analogs with human 11β-HSD1.
3D structures of diallyl BPA (red, DABPA, A), and dimethyl BPA (red, DMBPA, C), bisphenol A (positive control) (red, BPA, E), 4-nonyphenol (negative control) (red, 4-NP, G), which bind to cortisone (cyan) binding domain; 2D superimposed structures of DABPA (purple, B), DMBPA (purple, D), BPA (purple, F) and 4-NP (purple, H), which overlap with cortisone (cyan): circled red showing overlapping residues with cortisone, green line showing the hydrogen bonds.

A human 11β-HSD1

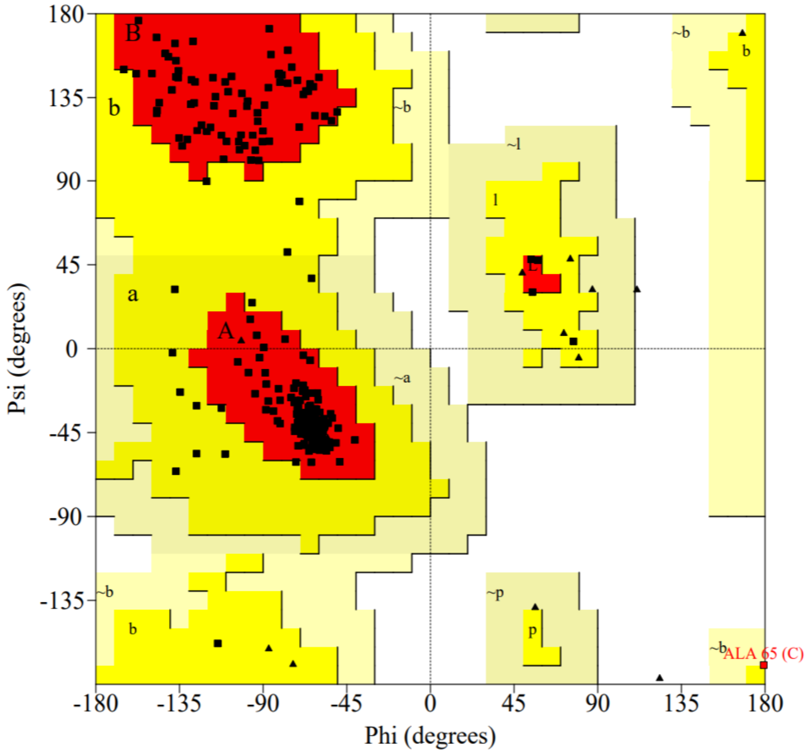

B Rat 11β-HSD1

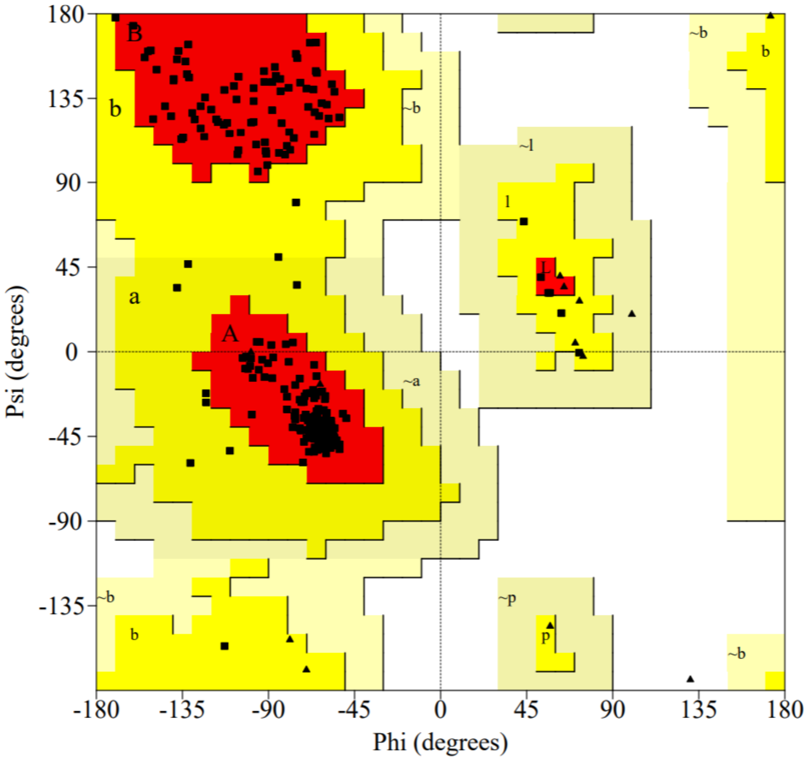

Figure S2. The Ramachandran figure illustrates the phi-psi torsion angles for human 11β-HSD1 (A) and rat 11β-HSD1 (B). The red regions represent the most desirable phi-psi value combinations. The white region represents an undesirable phi-psi combination.

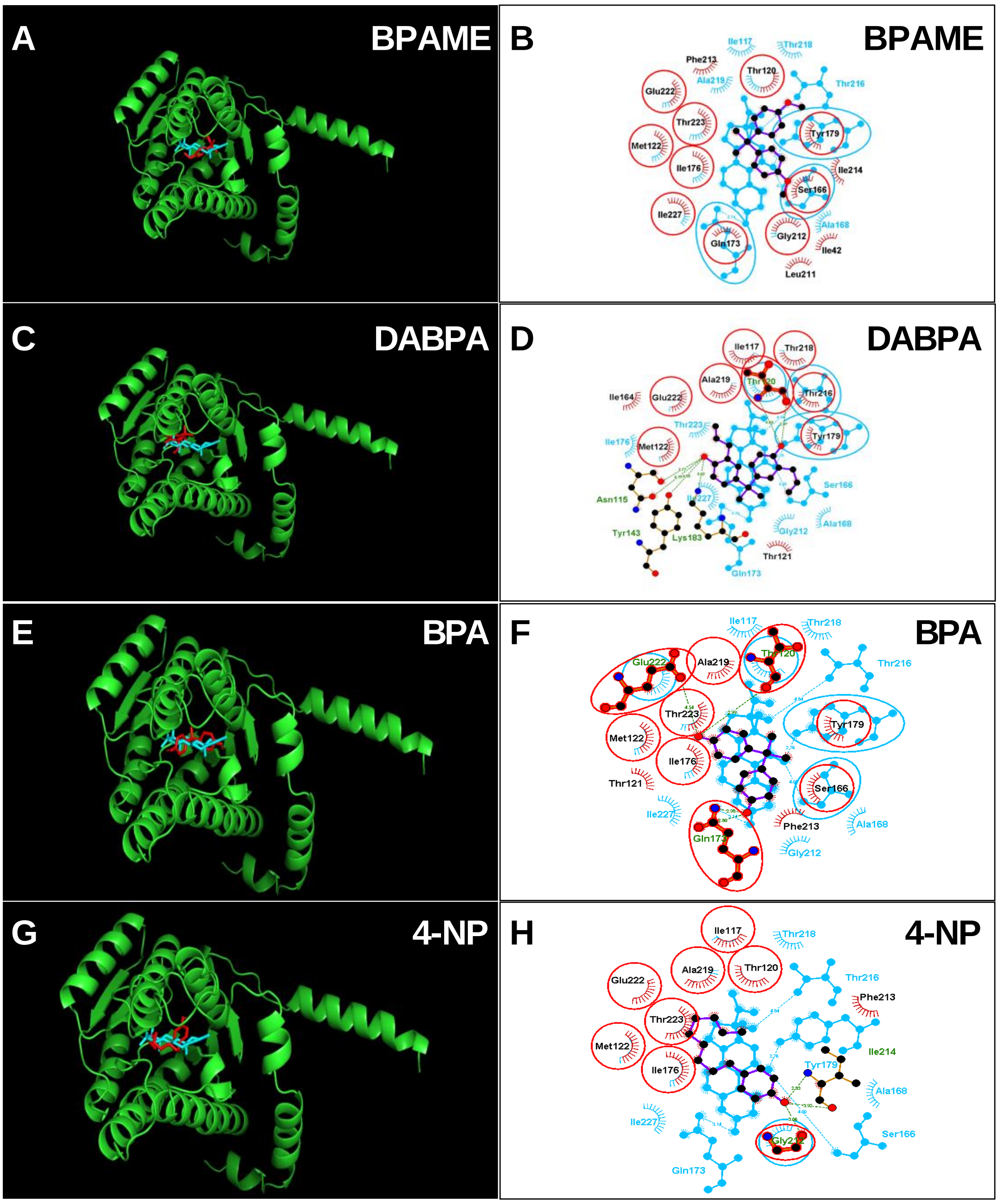

Figure S3. Molecular docking of bisphenol A analogs with rat 11β-HSD1.
3D structures of bisphenol A (BPA) dimethyl ether (red, BPAME, A), diallyl BPA (red, DABPA, C), bisphenol A (positive control) (red, BPA, E), 4-nonyphenol (negative control) (red, 4-NP, G), which bind to 11-dehydrocorticosterone (DHC, cyan) binding domain; 2D superimposed structures of BPAME (purple, B), DABPA (purple, D), BPA (purple, F), and 4-NP (purple, H), which overlap with DHC (cyan): circled red showing overlapping residues with cortisone, green line showing the hydrogen bonds.

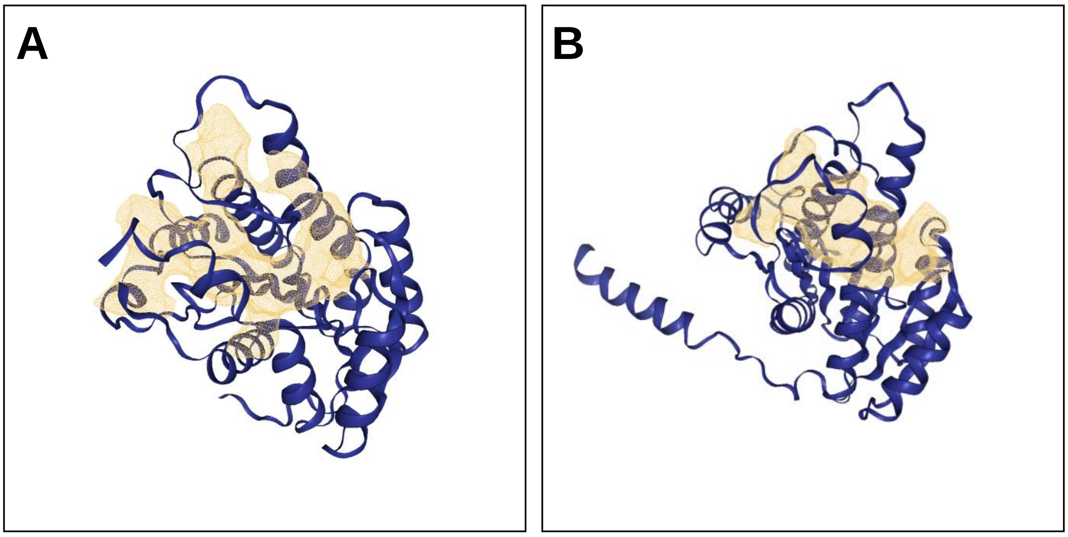

Figure S4. Binding cavity of human and rat 11β-HSD1 enzymes.
Binding cavity analysis was performed by Proteinplus web server for cortisol with human 11β-HSD1 (A) and 11-dehydrocorticosterone (DHC) with rat enzyme (B). Yellow area indicates the binding cavity. Apparently, human 11β-HSD1 has larger binding cavity than rat enzyme.

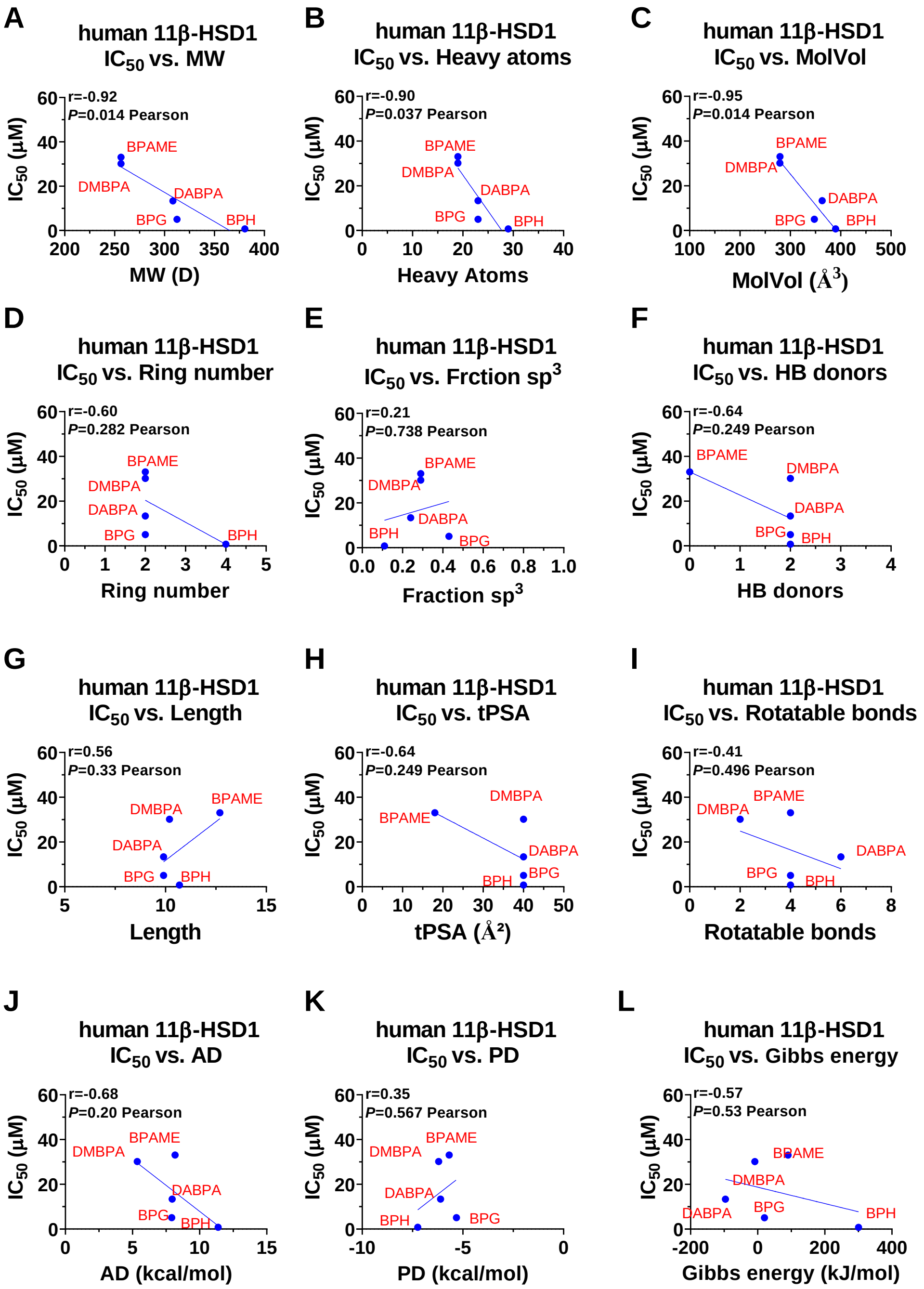

Figure S5. Bivariate correlation analysis for structural features of BPA analogues with IC_{50} values on human 11β-HSD1 enzyme.

The correlation analysis of BPA analogues on human 11β-HSD1 for molecular weight (MW, A), heavy atoms(B), molecular volume (C), ring number (D), fraction sp^3 (E), hydrogen bond (HB) donors (F), length (G), tPSA (H), rotatable bonds (I), apolar desolvation energy (AD, J), polar desolvation energy (PD, K) and Gibbs energy (L).

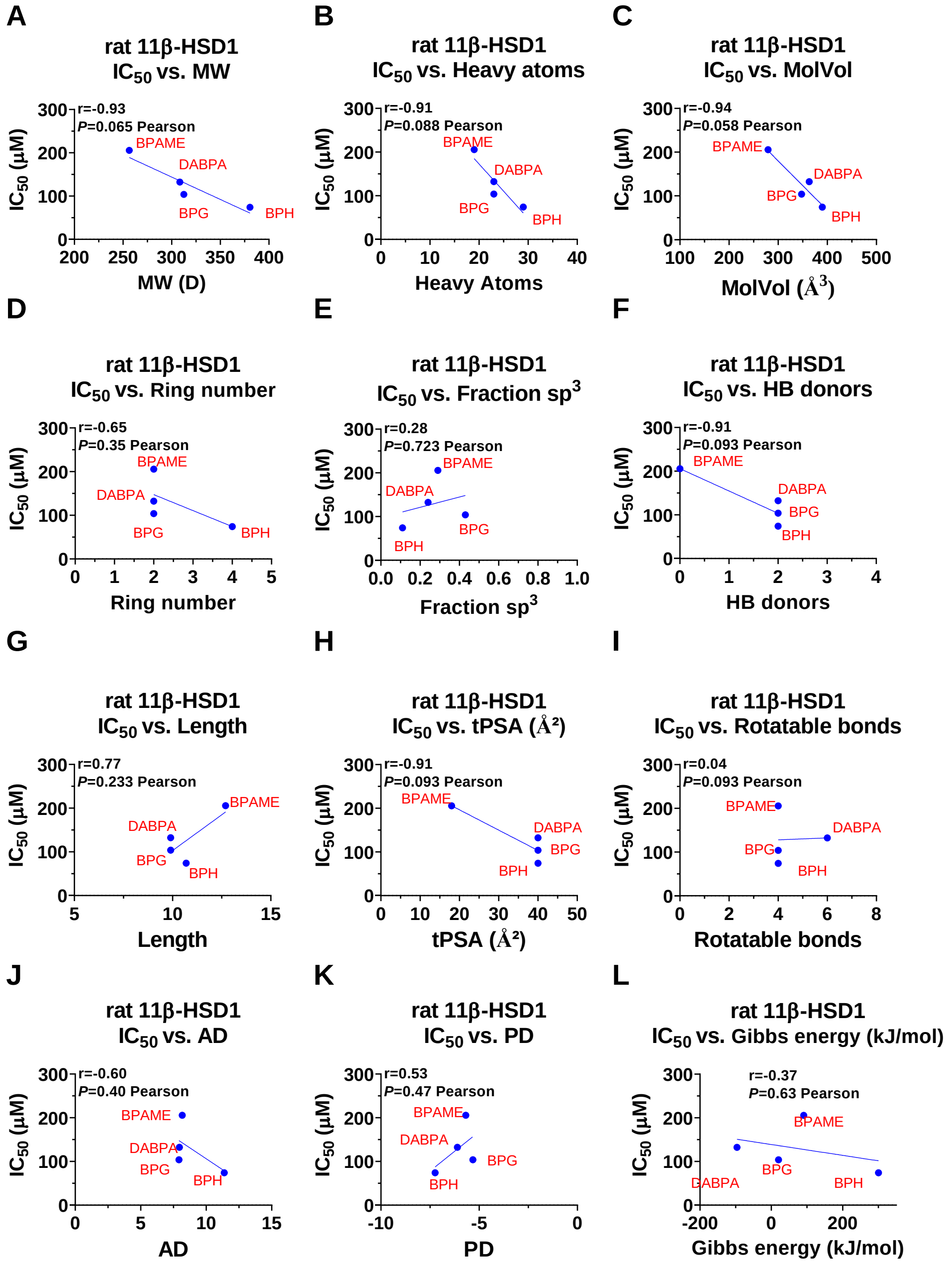

Figure S6. Bivariate correlation analysis for structural features of BPA analogues with IC_{50} values on rat 11β-HSD1 enzyme.

The correlation analysis of BPA analogues on rat 11β-HSD1 for molecular weight (MW, A), heavy atoms(B), molecular volume (C), ring number (D), fraction sp^3 (E), hydrogen bond (HB) donors (F), length (G), tPSA (H), rotatable bonds (I), apolar desolvation energy (AD, J), polar desolvation energy (PD, K) and Gibbs energy (L).

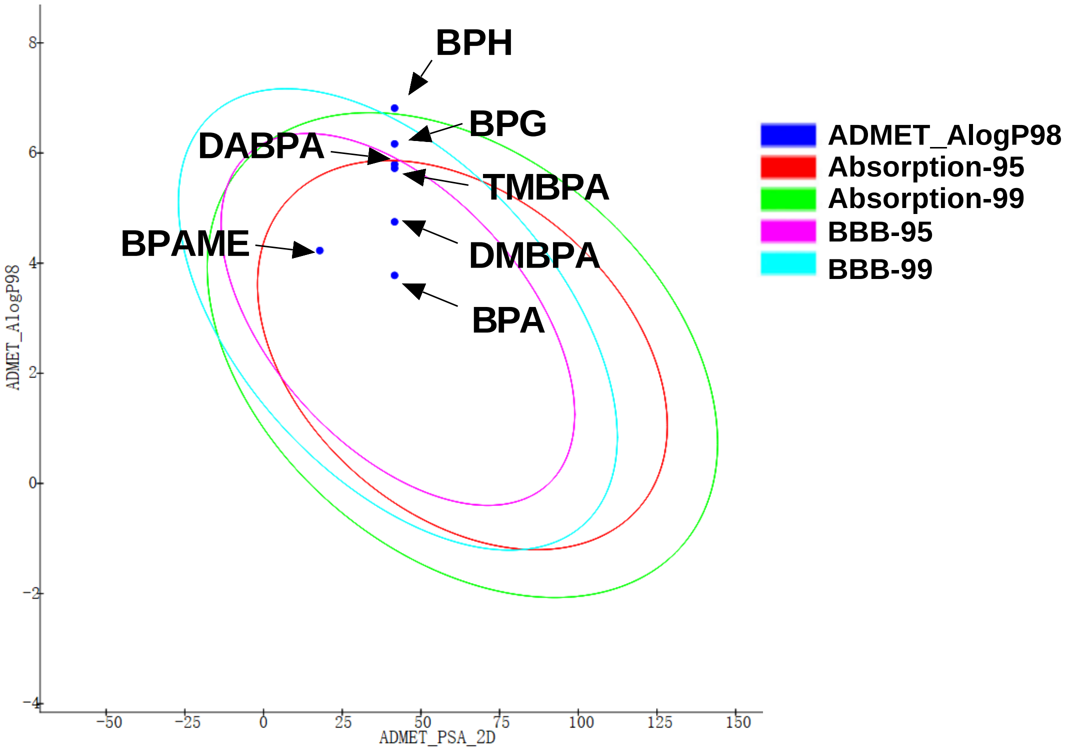

Figure S7. ADMET prediction of BPA analogues.

Plotted by ADMET_PSA_2D vs. ADMET_AlogP98. The dark blue dots represent AlogP98 of each drug. The red and green ellipses represent 95 and 99% confidence intervals of the blood–brain barrier (BBB) permeability model, respectively, and the rose red and light blue ellipses represent 95 and 99% confidence intervals of the human intestinal absorption (HIA) model, respectively.
